# Rectal-prolapse repair in men is safe, but outcomes are not well understood

**DOI:** 10.1093/gastro/goz016

**Published:** 2019-05-09

**Authors:** Vitaliy Y Poylin, Jennifer L Irani, Reza Rahbar, Muneera R Kapadia

**Affiliations:** 1 Beth Israel Deaconess Medical Center, Boston, MA, USA; 2 Brigham and Woman's Hospital, Boston, MA, USA; 3 UNC Health Care, Raleigh, NC, USA; 4 University of Iowa, Iowa City, IA, USA

**Keywords:** Rectal prolapse, surgical procedure, sexual function, men

## Abstract

**Introduction:**

Rectal prolapse is a condition that occurs infrequently in men and there is little literature guiding treatment in this population. The purpose of this study was to evaluate the surgical approach and outcomes of rectal-prolapse repair in men.

**Methods:**

A retrospective multicenter review was conducted of consecutive men who underwent rectal-prolapse repair between 2004 and 2014. Surgical approaches and outcomes, including erectile function and fecal continence, were evaluated.

**Results:**

During the study period, 58 men underwent rectal-prolapse repair and the mean age of repair was 52.7 ± 24.1 years. The mean follow-up was 13.2 months (range, 0.5–117 months). The majority of patients underwent endoscopic evaluation (78%), but few patients underwent anal manometry (16%), defecography (9%) or ultrasound (3%). Ten patients (17%) underwent biofeedback/pelvic-floor physical therapy prior to repair. Nineteen patients (33%) underwent a perineal approach (most were perineal proctosigmoidectomy). Thirty-nine patients (67%) underwent repair using an abdominal approach (all were suture rectopexy) and, of these, 77% were completed using a minimally invasive technique. The overall complication rate was 26% including urinary retention (16%), which was more common in patients undergoing the perineal approach (32% vs. 8%, *P* = 0.028), urinary-tract infection (7%) and wound infection (3%). The overall recurrence rate was 9%, with no difference between abdominal and perineal approaches. Information on sexual function was missing in the majority of patients  both before and after surgery (76% and 78%, respectively).

**Conclusion:**

Rectal-prolapse repair in men is safe and has a low recurrence rate; however, sexual function was poorly recorded across all institutions. Further studies are needed to evaluate to best approach to and functional outcomes of rectal-prolapse repair in men.

## Introduction

Full-thickness rectal prolapse is a benign, but stressful, condition that leads to problems with bleeding, fecal incontinence and obstructed defecation [1–8]. Rectal prolapse can significantly affect quality of life and therefore should be repaired whenever possible. Older women are the most commonly affected group. Multiple risks factors have been proposed for developing rectal prolapse, with the anatomy of the female pelvis, child-bearing and constipation thought to be the most important factors for developing this condition [1, 2]. For this reason, rectal prolapse in men is less common and not well understood. It is thought to be more common in patients suffering from constipation, but reliable data are missing. A number of guidelines exist in both colorectal as well as urogynecological literature with a proposed work-up and approach to female patients with this condition and comprehensive work-up is generally recommended, likely in part because of the known higher rates of other pelvic-floor problems and in part due to more comprehensive studies of these problems in female patients. Generally, rectal prolapse in men is treated in the same fashion as in female patients, but no reliable studies exist to guide the proper care for these patients.

Two major approaches are currently used to correct rectal prolapse. The perineal approach, which has been traditionally reserved for older and debilitated patients, has been reported to be generally better tolerated with a lower rate of complications; however, this comes at the price of higher rates of recurrence and functional changes (e.g. urgency and frequency) [1, 2, 9, 10]. Abdominal approaches carry a lower rate of recurrence, but often entail a higher complication rate [1, 3, 4, 9, 10]. With minimally invasive abdominal approaches, there tends to be quicker recovery, less pain, shorter hospital stays and a lower complication rate [5–7, 11]. In addition to typical post-operative concerns such as infection, bleeding, recurrence and change in fecal continence, men undergoing pelvic surgery are more likely to develop urinary and sexual dysfunction (e.g. retrograde ejaculation and impotence) [8]. Outcomes following abdominal or perineal approaches to rectal prolapse in men are not well understood. Therefore, the purpose of this study was to better understand the male-patient cohort who present with rectal prolapse, and evaluate the surgical approach and outcomes of rectal-prolapse repair in men.

## Methods

A retrospective multicenter review of consecutive men who underwent rectal-prolapse repair between 2004 and 2014 was conducted at the following institutions: Beth Israel Deaconess Medical Center (Boston, MA), Brigham and Woman’s Hospital (Boston, MA), University of Iowa Hospitals and Clinics (Iowa City, IA) and University of North Carolina Medical Center (Chapel Hill, NC). Patients were identified from existing databases and current procedural terminology code searches. Variables collected included demographics, comorbidities, surgical approach and outcomes, including erectile function and fecal continence. There were no exclusion criteria for this study. This study was approved by the Institutional Review Board at Beth Israel Deaconess Medical Center and extended to other institutions. The procedure performed as well as the pre-operative evaluation and post-operative follow-up were at the discretion of the surgeon and institution.

### Statistical analysis

All analyses were conducted using IBM SPSS Statistics version 21.0.0 for Macintosh (IBM Corp., Armonk, NY). Descriptive statistics were utilized to examine the patient cohort. Throughout all analyses, statistical significance was determined by a criterion of *P*-value less than or equal to 0.05.

## Results

During the study period, 58 men underwent rectal-prolapse repair and the mean age of repair was 52.7 ± 24.1 years. Eight patients (14%) resided in long-term care facilities and 13 (22%) had concurrent psychiatric diagnoses. Prior to surgery, the majority of patients (*n* = 45, 78%) underwent endoscopic evaluation, but few patients underwent anal manometry (*n* = 9, 16%), defecography (*n* =5,  9%) or ultrasound (*n* =2,  3%). Ten patients underwent biofeedback/pelvic-floor physical therapy prior to repair.

Nineteen patients (33%) underwent a perineal approach (most were perineal proctosigmoidectomy). Thirty-nine patients (67%) underwent repair using an abdominal approach (all included suture rectopexy, some with sigmoid colectomy in addition) and, of these, 77% were completed using a minimally invasive technique. Patients who underwent an abdominal operation were younger (median age: 45 vs. 72 years, *P* < 0.05) and had a lower ASA (American Society of Anesthesiologists) score (*P* < 0.001) compared to patients who underwent a perineal operation. There was no difference in types of procedures performed between participating institutions.

The overall complication rate was 26% (*n* = 15), including urinary retention (*n* = 9, 16%), which was more common in patients undergoing the perineal approach (32% vs. 8%, *P* = 0.028), urinary-tract infection (*n* = 4, 7%) and wound infection (*n* = 2, 3%). The overall recurrence rate was 9%, with no difference between the abdominal and perineal approaches over a mean follow-up period of 13.2 months (range, 0.5–117 months).

**Figure 1.**
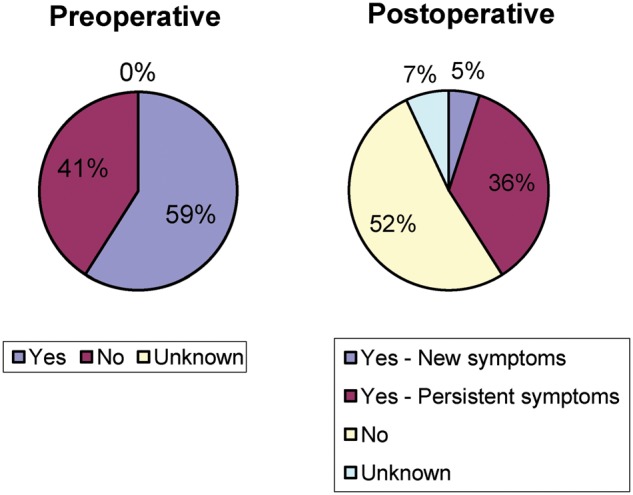
Constipation before and after surgery

**Figure 2.**
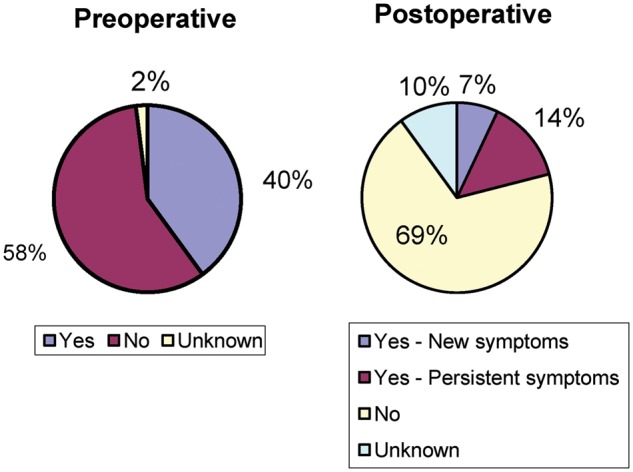
Fecal incontinence before and after surgery

**Figure 3.**
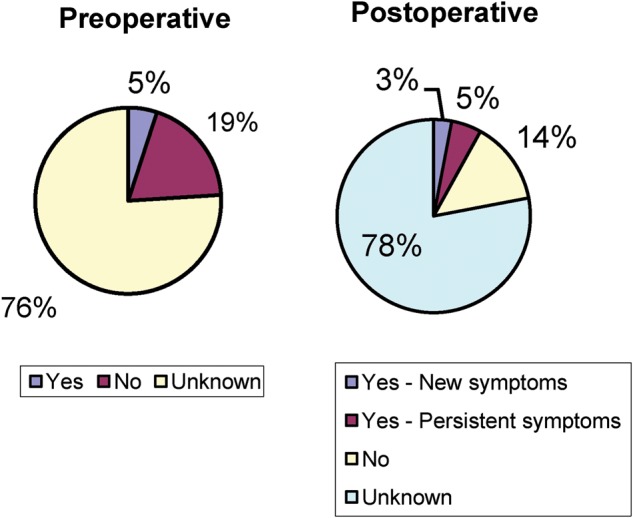
Sexual function before and after surgery

There was a decrease in post-operative constipation in patients who reported having problems prior to surgery (36% from 59%) and 5% of patients reported new onset of constipation, with data missing in 7% of patients (Figure 1). Rates of fecal incontinence similarly decreased from 40% to 14%, but 7% of patients developed new symptoms of incontinence and data were missing in 2% of patients prior to and 10% of patients after surgery (Figure 2). Sexual function was not documented in 76% of patients prior to surgery and 78% of patients after surgery, and 3% of patients reported new onset of sexual dysfunction after surgery (Figure 3).

## Discussion

Rectal prolapse in men is a rare condition and overall comprises only a small proportion of rectal-prolapse cases [9–11]. The focus of this study was to examine rectal prolapse in the male population and evaluate surgical options for the repair of rectal prolapse in this population. Over a 10-year period at four large academic medical centers, only 58 male patients who underwent rectal-prolapse repair were identified. Endoscopic evaluation was common, but most patients underwent little other pre-operative testing. Two-thirds of the patients underwent an abdominal suture rectopexy and most were performed in a minimally invasive fashion. The overall recurrence and complication rates were low, and both perineal and abdominal approaches are generally well tolerated by patients. This is different compared to what has been seen in studies of female patients and may be due to the low numbers in this study. The vast majority of abdominal cases were performed with a minimally invasive approach, which mirrors the overall trend in rectal-prolapse repair.

Although pelvic-floor dysfunction is rare in men, very few patients had proper pelvic-floor work-up or an attempt to treat (biofeedback/pelvic-floor physical therapy) pelvic-floor symptoms. Reasoning for this was not well recorded, so it is impossible to say whether this population of patients has higher rates of pelvic-floor dysfunction compared to normal men. This study also showed improvement in both constipation as well as fecal incontinence, in these patients, with only a few developing new symptoms in each category. It is likely that some of the initial problems with constipation and incontinence were related to a prolapsing rectum causing obstructed defecation and producing discharge (often interpreted by patients as incontinence). However, very few functional data were collected on these patients before or after surgery, with a uniform lack of use of validated instruments, making future decision-making and discussion with these patients very difficult. The most striking result of this study is a lack of documentation of sexual function before and after surgery in the majority of the patients. Although pelvic surgery is associated with both retrograde ejaculation and impotence, it was recorded only in 24% of patients prior to surgery and 22% of patients after surgery (including 3% of new symptoms).

The main limitations in this study include its retrospective nature and small study population. In addition, there is also variability in the operations performed. Due to the rarity of this problem and its retrospective nature, we could not control for the type of procedure performed, pre- or post-operative examinations or length of follow-up, all of which could have influenced some of the outcomes. This indicates a critical need for better standardization in work-up, surgery and follow-up for men with rectal prolapse.

The current approach to pelvic-floor dysfunction and prolapse is continuing to evolve with the popularization of ventral rectopexy as well as robotic approaches, making a minimally invasive approach easier in the confines of the narrow male pelvis. A combination of the above has been shown to be beneficial in women with similar problems, both in surgical outcomes as well as in minimizing functional issues after surgery, suggesting that the same may be true in men, although these recent additions are not well studied or understood in the male population and warrant further investigation.

In conclusion, rectal-prolapse repair in men is safe and has a low recurrence rate; however, sexual function was poorly recorded across all institutions. Pre-operative evaluation was most often limited to physical examination and colonoscopy. Further studies are needed to evaluate the best approach to and functional outcomes of rectal-prolapse repair in men.

## Authors’ contributions

V.Y.P.: concept, data collection, data analysis, manuscript preparation and review. J.L.I.: concept, data collection, manuscript review. R.R.: concept, data collection, manuscript review. M.R.K.: concept, data collection, data analysis, manuscript preparation and review.

## Funding

None.
